# Phenotype-Guided Management of Atrial Fibrillation in Heart Failure: From Rate Control to Catheter Ablation

**DOI:** 10.3390/jcm15176519

**Published:** 2026-08-23

**Authors:** Ebru Şahin, İsa Ardahanlı, Onur Akhan, Ramazan Aslan, Mustafa Kaplangöray

**Affiliations:** 1 Department of Cardiology, Memorial Ankara Hospital, Ankara 06520, Türkiye; 2 Department of Cardiology, Faculty of Medicine, Bilecik Şeyh Edebali University, Bilecik 11230, Türkiye; isaardahanli@gmail.com; 3 Department of Cardiology, Sancaktepe Şehit Prof. Dr. İlhan Varank Training and Research Hospital, Istanbul 34785, Türkiye; akhanonur@gmail.com; 4 Department of Cardiology, Bilecik Training and Research Hospital, Bilecik 11040, Türkiye; drramazanaslan11@gmail.com; 5 Department of Cardiology, Acıbadem Eskişehir Hospital, Eskişehir 26130, Türkiye; mkaplangoray@gmail.com

**Keywords:** atrial fibrillation, heart failure, HFrEF, HFmrEF, HFpEF, rhythm control, rate control, catheter ablation, arrhythmia-induced cardiomyopathy, pulmonary vein isolation

## Abstract

Atrial fibrillation (AF) and heart failure (HF) frequently coexist, but the clinical relevance of AF may differ according to whether it appears to be a potentially reversible contributor, an aggravating factor in established HF, or a marker of advanced substrate. The driver–modifier–marker lens used in this review is a provisional, nonvalidated aid to clinical reasoning and should not be interpreted as a treatment score. This narrative review was informed by dated searches of PubMed/MEDLINE, the Cochrane Library, and OpenAlex through 29 July 2026, followed by a targeted update on 30 July 2026. It considers HF with reduced, mildly reduced, and preserved ejection fraction together with AF timing, burden, ventricular-rate exposure, myocardial substrate, and reversibility. Early rhythm control may be particularly relevant when AF is recent or temporally associated with ventricular dysfunction, symptoms, decompensation, or inadequate cardiac resynchronization therapy delivery. Evidence supporting catheter ablation is most direct in suspected AF-mediated cardiomyopathy and selected HFrEF populations, whereas evidence in HFpEF more consistently supports symptom relief, improved exercise hemodynamics, and AF-burden reduction than mortality reduction. Pulmonary vein isolation remains the procedural foundation. Radiofrequency, cryoballoon, and pulsed-field ablation are effective in broad AF populations, but HF phenotype-specific prognostic superiority has not been established for any energy source. Clinical decisions should reflect the design and directness of the evidence together with expected benefit, rhythm durability, procedural risk, patient-reported outcomes, stroke prevention, guideline-directed HF therapy, risk-factor management, and patient preference.

## 1. Introduction

Atrial fibrillation (AF) and heart failure (HF) frequently coexist and reinforce one another. HF promotes AF through atrial pressure and volume overload, chamber stretch, neurohormonal activation, inflammation, and fibrosis, whereas AF may worsen HF through rapid or irregular ventricular activation, loss of atrial contraction, and atrioventricular dyssynchrony. Accordingly, AF may be a principal cause of potentially reversible myocardial dysfunction, an aggravating factor in established HF, or a manifestation of advanced atrial and ventricular disease [1,2,3].

Earlier pharmacological rate-versus-rhythm trials addressed a different treatment context from contemporary early-rhythm-control and ablation trials. In the Atrial Fibrillation and Congestive Heart Failure (AF-CHF) trial, 1376 patients with a left ventricular ejection fraction (LVEF) of 35% or less were assigned to predominantly drug-based rhythm control or rate control, and cardiovascular mortality did not differ significantly (27% versus 25%; hazard ratio [HR] 1.06; *p* = 0.59) [4]. This result should not be interpreted as evidence that lowering AF burden is uniformly ineffective: durable sinus rhythm was difficult to maintain, antiarrhythmic drug exposure was substantial, and later studies evaluated earlier intervention or catheter ablation in different populations with different monitoring methods and endpoints [5,6,7,8,9,10,11].

Previous reviews and guidelines distinguish AF-induced cardiomyopathy, in which AF is the principal cause of left ventricular (LV) dysfunction, from AF-mediated cardiomyopathy, in which AF aggravates structural disease. This review uses driver, modifier, and marker only as descriptive terms within HFrEF, HFmrEF, and HFpEF; they are not a new classification and do not determine treatment. Instead, they frame whether AF-burden reduction is likely to help and whether durable reduction is achievable [2,12,13].

## 2. Literature Search Strategy for This Narrative Review

### 2.1. Information Sources and Search Dates

This narrative review was informed by structured searches of PubMed/MEDLINE and the Cochrane Library, including the Cochrane Database of Systematic Reviews (CDSR) and the Cochrane Central Register of Controlled Trials (CENTRAL), from database inception through 29 July 2026. OpenAlex was searched from 1 January 1900 through 29 July 2026 as a supplementary multidisciplinary source. A targeted update on 30 July 2026 identified newly indexed and online-first reports relevant to ablation strategy, pulsed-field ablation, and heart-failure phenotypes. Reference lists of pivotal trials, guidelines, consensus documents, and major reviews were examined, and forward citation links were used to identify additional reports. The exact database syntax and dated yields are reported in Supplementary Tables S1 and S2; dated machine-readable verification exports, query text, API parameters, and deduplication files are provided in the separate Supplementary Data S1 archive.

### 2.2. Search Concepts and Supplementary Identification

Database-specific subject headings and free-text terms covered three concepts: atrial fibrillation; heart-failure phenotype; and clinical management. The phenotype block included the abbreviations HFrEF, HFmrEF, and HFpEF together with the full terms heart failure with reduced, mildly reduced, preserved, or improved ejection fraction and arrhythmia- or tachycardia-induced cardiomyopathy. Management terms included rate control, early or conventional rhythm control, antiarrhythmic drugs, cardioversion, catheter ablation, pulmonary vein isolation, radiofrequency, cryoballoon and pulsed-field energy, additional lesion sets, atrioventricular-junction ablation, cardiac resynchronization therapy, and anticoagulation. PubMed/MEDLINE used MeSH and title/abstract fields with an evidence-type block and an animal-only exclusion. The Cochrane and OpenAlex strategies were adapted to their respective search interfaces without changing the three core concepts. The dated OpenAlex record set used the operational field-restricted title_and_abstract.search filter. Current OpenAlex documentation labels field-suffixed search filters as legacy and recommends the separate search = parameter. Supplementary Table S1 therefore preserves the archival syntax for provenance and also reports the current supported parameterization. Because the current search = parameter searches titles, abstracts, and available full text, it is documented as a sensitivity and future-rerun specification rather than being treated as field-identical to, or retrospectively substituted for, the dated title-and-abstract set.

Supplementary topic-specific searches and citation chaining addressed AF-mediated cardiomyopathy, quantified AF burden and continuous monitoring, cardiac magnetic resonance and fibrosis, timing of ablation, quality of life and other patient-reported outcomes, access to care and social determinants, guideline-directed heart-failure therapy, conduction-system pacing, cardiometabolic risk-factor management, additional lesion sets, hybrid ablation, and platform-specific PFA evidence. ClinicalTrials.gov was checked by trial name or NCT identifier. Registry entries were used only to describe study design and status, not to infer unreported efficacy.

### 2.3. Eligibility and Evidence Scope

Reports were eligible for the principal synthesis when they included adults with atrial fibrillation and a defined heart-failure phenotype or an extractable heart-failure subgroup and evaluated a management strategy relevant to the review question. Eligible outcomes included mortality, heart-failure hospitalization or urgent care, stroke or systemic embolism, AF recurrence or burden, left ventricular ejection fraction, exercise capacity, symptoms, quality of life, treatment burden, and procedural safety. Priority was given to contemporary guidelines and consensus statements, randomized trials, prespecified heart-failure subgroup analyses, systematic reviews and meta-analyses, and comparative observational studies when higher-level evidence was unavailable.

Pediatric and preclinical studies, case reports, conference abstracts without adequate outcome data, duplicate reports, small uncontrolled series without useful comparative information, and studies without an AF–HF population or extractable HF subgroup were not used as principal evidence. Single-arm pivotal PFA studies and large safety registries were retained when they addressed platform-specific effectiveness, workflow, or uncommon adverse events that could not be assessed adequately in randomized HF trials. Their design limitations are stated in the text and tables.

### 2.4. Record Handling and Narrative Synthesis

The dated searches retrieved 819 PubMed/MEDLINE records, five CDSR reviews, 1454 CENTRAL records, and 3744 OpenAlex records after within-source deduplication. Matching PubMed and OpenAlex by PMID, digital object identifier, and normalized title identified 558 overlaps and yielded 4005 unique records across those two sources before relevance prioritization. CENTRAL was retained as a separate source because complete bulk export was not available in the access environment; it was therefore not included in the PubMed–OpenAlex cross-database deduplication. These figures describe dated retrieval and deduplication rather than a formal study-selection flow.

This was a critical narrative review rather than a systematic review of a single intervention. Evidence was selected purposively for clinical relevance, authority, and applicability to AF–HF phenotypes. Duplicate independent screening, protocol registration, a formal risk-of-bias instrument, quantitative pooling, and a PRISMA-style study-selection flow were not undertaken. The reported search yields describe retrieval and deduplication, not the denominator of a formally screened evidence set. Differences in population selection, HF definition, rhythm monitoring, endpoint construction, background therapy, ablation platform, and follow-up were considered before clinical interpretation. Supplementary Table S3 summarizes this evidence-handling framework.

To make evidentiary provenance visible, studies are labeled in the text and tables as direct randomized evidence, prespecified randomized subgroup evidence, post hoc subgroup evidence, observational or single-arm evidence, pilot evidence, guideline/consensus support, or author-derived interpretation. Direct randomized evidence in the target phenotype is given the greatest weight; prespecified subgroups are interpreted more strongly than post hoc analyses; and observational, single-arm, and pilot findings are treated as hypothesis-generating. These design labels are not a formal GRADE certainty assessment.

A 2026 review of atrial cardiomyopathy was added as contextual evidence [14]. It was not included in the dated retrieval counts and was not treated as intervention-effect evidence.

## 3. Pathophysiology and Phenotype-Based Assessment

### 3.1. The Bidirectional Relationship Between Atrial Fibrillation and Heart Failure

Heart failure promotes atrial fibrillation through left-atrial pressure and volume overload. Chamber stretch, dilatation, interstitial fibrosis, altered conduction, neurohormonal activation, and functional mitral regurgitation create a substrate in which pulmonary-vein triggers are more likely to produce sustained arrhythmia. Ventricular myocardial disease and secondary mitral regurgitation are prominent contributors in HFrEF, whereas atrial myopathy, obesity, hypertension, renal dysfunction, and systemic inflammation are more prominent in HFpEF [2]. Pierucci et al. describe atrial cardiomyopathy as a multidimensional substrate involving structural, electrical, mechanical, and molecular remodeling. This framework may help contextualize the relationships among systemic risk factors, HF, AF persistence, thromboembolism, and recurrence; however, it does not provide a validated treatment-selection or ablation score [14].

Atrial fibrillation may worsen heart failure despite an apparently acceptable resting ventricular rate. Loss of atrial contraction is particularly important in HFpEF; irregular ventricular activation and high arrhythmia burden can reduce ventricular efficiency across the ejection-fraction spectrum; and persistent tachycardia can produce a reversible cardiomyopathy. Adequate resting rate control therefore does not exclude AF-mediated ventricular dysfunction [15].

### 3.2. HF Phenotypes and Mechanisms of AF Development

For comparability with contemporary trials and treatment guidelines, this review reports HFrEF as LVEF ≤ 40%, HFmrEF as LVEF 41–49%, and HFpEF as LVEF ≥ 50% accompanied by objective evidence of heart failure, including structural or functional cardiac abnormalities, raised filling pressure, natriuretic peptides, or congestion [16,17]. The 2026 Second Universal Definition moves away from rigid ejection-fraction thresholds and emphasizes reduced, preserved, and improved ejection fraction together with etiology and clinical trajectory. Accordingly, the EF categories used here are descriptive trial-alignment tools rather than stand-alone diagnoses or treatment rules [18].

#### 3.2.1. Heart Failure with Reduced Ejection Fraction (HFrEF; LVEF ≤ 40%)

In HFrEF, increased filling pressure, functional mitral regurgitation, and neurohormonal activation drive left-atrial remodeling. Once AF develops, rapid or irregular ventricular activation and loss of atrioventricular synchrony can further reduce cardiac output. The clinically important distinction is between AF caused by established ventricular disease and AF-mediated cardiomyopathy, in which AF burden is a major reversible component of LV dysfunction [2,11,15].

#### 3.2.2. Heart Failure with Mildly Reduced Ejection Fraction (HFmrEF; LVEF 41–49%)

Heart failure with mildly reduced ejection fraction (HFmrEF) is a heterogeneous intermediate phenotype rather than a single trajectory. Previous HFrEF with partial recovery, an ejection-fraction decline after AF onset, or limited ventricular scar may indicate a reversible AF-related component. By contrast, progressive cardiometabolic disease with marked atrial remodeling may resemble HFpEF. Previous LVEF, the temporal relation between AF and HF deterioration, ventricular scar, and the response to rhythm restoration may therefore provide more therapeutic information than the current LVEF value alone [16,17,18].

#### 3.2.3. Heart Failure with Preserved Ejection Fraction (HFpEF; LVEF ≥ 50%)

Heart failure with preserved ejection fraction (HFpEF) is characterized by a stiff ventricle, abnormal exercise filling pressure, atrial myopathy, and a high burden of obesity, hypertension, diabetes, kidney disease, and sleep apnea. Loss of atrial contraction and ventricular irregularity can markedly increase pulmonary capillary wedge pressure without changing EF. In advanced HFpEF, however, AF may be one manifestation of diffuse atrial and ventricular disease rather than the primary cause of symptoms [2,3,19].

### 3.3. Distinct Clinical Dimensions and Descriptive Roles of AF

For the purposes of this review, five clinical dimensions are considered separately before applying a causal label: (1) AF temporal pattern and its relation to HF deterioration; (2) quantified AF burden; (3) ventricular-rate burden, including irregularity and exercise rate; (4) atrial and ventricular substrate, including chamber size, fibrosis, valve disease, and competing cardiomyopathy; and (5) reversibility after rhythm restoration or substantial burden reduction. These variables are related but not interchangeable. A patient may have persistent AF with low ventricular-rate burden, extensive atrial disease with modest measured AF burden, or substantial ventricular recovery despite recurrent short episodes.

Within this proposed framework, driver, modifier, and marker are used only as working interpretations after the five dimensions have been considered. They are not diagnoses, fixed phenotypes, or a validated score, and more than one role may coexist. The interpretation considers whether AF preceded deterioration, whether burden and ventricular-rate exposure may be clinically relevant, whether a stronger myocardial or valvular cause is present, and what changes occur after rhythm restoration or substantial burden reduction [2,15].

A driver-predominant presentation is suspected when AF precedes otherwise unexplained LV dysfunction and a high verified burden is accompanied by limited competing disease. A modifier role is more likely when established HF worsens during AF through higher filling pressure, functional mitral regurgitation, reduced exercise capacity, recurrent congestion, or impaired cardiac resynchronization therapy (CRT) delivery. A marker-predominant presentation describes long-standing AF accompanying advanced atrial, ventricular, valvular, pulmonary vascular, or systemic disease; rhythm control may still improve symptoms, but durable rhythm is less likely [12,20].

The working role must be reassessed after HF optimization and rhythm intervention. Ventricular recovery or a reproducible clinical response with verified AF-burden reduction strengthens a driver or modifier interpretation. Persistent dysfunction or symptoms despite a major burden reduction increase the weight of the underlying HF substrate. The proposed frameworksets out this reassessment explicitly; the categories remain overlapping and unvalidated [11,21,22].

## 4. Phenotype-Guided Treatment: From Rate Control to Early Ablation

### 4.1. Two Questions Before Choosing Treatment

Two questions structure rhythm-control selection: whether reducing AF burden is likely to improve ventricular function, HF events, exercise capacity, or symptoms, and whether the selected intervention is likely to achieve a durable burden reduction. These questions should be assessed after, or in parallel with, correction of hemodynamic instability and congestion, initiation of phenotype-directed HF therapy, stroke prevention, and treatment of reversible precipitants [1,16,17]. Recent AF, clinical deterioration after AF onset, high burden, smaller atrial size, limited ventricular scar, and a previous response to sinus rhythm favor both benefit and durability. Long-standing AF, advanced remodeling, valve disease, pulmonary hypertension, frailty, renal dysfunction, obesity, and untreated sleep apnea reduce expected durability without, in isolation, establishing futility [23].

The prespecified EAST-AFNET 4 heart-failure analysis provides the rationale for acting before the substrate becomes fixed. Among 798 patients with recent AF and HF, including 442 with HFpEF, 211 with HFmrEF, and 132 with HFrEF among those with classified LVEF, early rhythm control reduced the composite of cardiovascular death, stroke, or hospitalization for worsening HF or acute coronary syndrome from 7.9 to 5.7 events per 100 patient-years over 5.1 years (hazard ratio [HR] 0.74, 95% confidence interval [CI] 0.56–0.97). Because the strategy combined antiarrhythmic drugs, cardioversion, and ablation, the result supports timely rhythm control across EF categories rather than immediate ablation for every patient [6].

### 4.2. Measuring AF Burden and Defining Treatment Success

Atrial fibrillation burden is the proportion of monitored time spent in AF. Episode number and duration, ventricular-rate distribution, and the monitoring window add information that a simple paroxysmal or persistent label cannot provide. A binary recurrence endpoint based on any atrial tachyarrhythmia lasting at least 30 s standardizes ablation studies, but it treats one short episode and sustained recurrence as equivalent [20,24].

Measurement depends on the device and observation period. Pacemakers, defibrillators, and implantable loop recorders can provide continuous burden and rate data. Holter and patch recordings sample a limited interval, while consumer wearables are often intermittent and depend on adherence and algorithm performance. Symptom-guided follow-up alone is inadequate because asymptomatic AF and symptoms without AF are common. Monitoring intensity should therefore match the decision: continuous device data are most informative when cardiomyopathy, CRT delivery, or a burden-outcome relation is being assessed [1,20,24].

The CASTLE-AF burden analysis illustrates why this distinction matters in HF. Among 280 patients in an as-treated analysis, the first post-ablation recurrence lasting more than 30 s was not related to mortality or HF hospitalization. By contrast, an AF burden below 50% at six months was associated with fewer primary outcome events (HR 0.33, 95% CI 0.15–0.71) and lower all-cause mortality (HR 0.23, 95% CI 0.07–0.71) [25]. This as-treated association does not validate 50% as a universal target; it shows that quantified burden can align with HF outcomes better than a single short recurrence.

Treatment success should be defined by the clinical objective rather than rhythm status alone. In suspected AF-mediated cardiomyopathy, AF burden should be interpreted with serial LVEF, ventricular volumes, biomarkers, and symptoms; in HFrEF, HF events and functional capacity should also be assessed; in HFpEF, symptoms, exercise tolerance, quality of life, and decompensation are central; and in CRT recipients, effective biventricular pacing must be documented. Validated patient-reported measures should be reported separately from electrophysiological recurrence. Persistent fatigue, anxiety, treatment concerns, or functional limitation may remain despite low AF burden. A recent patient-perspective article highlights this outcome gap, but as an editorial, it informs the outcome selection rather than comparative treatment efficacy [26].

### 4.3. Suspected AF-Mediated Cardiomyopathy: AF as a Major Contributor

When AF precedes otherwise unexplained LV dysfunction, the treatment goal is myocardial recovery, not symptom control alone. In CAMERA-MRI, 68 patients with persistent AF and LVEF ≤ 45% despite optimized rate control were randomized to ablation or continued medical rate control. At six months, LVEF increased by 18 ± 13% after ablation and by 4.4 ± 13% with rate control; normalization occurred in 58% and 9%, respectively. This magnitude of recovery, despite adequate baseline rate control, supports AF-burden reduction as both a therapeutic intervention and a retrospective test of causality [11,15].

Cardiac magnetic resonance (CMR) refines the expected response rather than creating a binary eligibility rule. In CAMERA-MRI, the absence of ventricular late gadolinium enhancement identified greater recovery. CAMERA-MRI II then enrolled 80 patients with LVEF ≤ 45% and at least 5% ventricular fibrosis: at 12 months, median LVEF increased by 20% with ablation and 4% with rate control (*p* < 0.001). Improvement was smaller when scar burden was ≥20%, but fibrosis did not eliminate benefit. Scar burden should therefore inform the anticipated magnitude of recovery and the discussion of procedural value, not serve as an automatic exclusion criterion [27].

For this phenotype, prompt cardioversion can test reversibility, while recurrent AF or persistent high burden favors early pulmonary vein isolation (PVI)-based ablation. Recovery, however, should not be equated with cure. In WITHDRAW-AF, 60 highly selected patients with previous persistent AF, normalized LVEF, and sustained sinus rhythm—97% after ablation—underwent staged HF-therapy withdrawal; 90% maintained cardiac magnetic resonance LVEF ≥ 50% at six months compared with 100% during continued therapy. In DEFINITION-AF, HF deterioration occurred in 3 of 23 patients assigned to guideline-directed medical therapy (GDMT) withdrawal and in none of 24 assigned to continuation; function recovered after treatment was restarted. These small pilot trials support monitored, individualized de-escalation in exceptional patients, but they do not justify routine withdrawal of HF therapy after apparent recovery [21,22].

### 4.4. HFrEF and HFmrEF: AF-Related Clinical Deterioration

In HFrEF, the most persuasive outcome data come from patients in whom AF remained clinically important and rhythm reduction was measurable. AATAC showed that ablation was superior to amiodarone in device-treated patients with persistent AF and LVEF < 40%, with freedom from AF in 70% versus 34%, hospitalization in 31% versus 57%, and mortality in 8% versus 18% over two years. CASTLE-AF extended this signal to hard outcomes: among 363 patients with symptomatic AF, LVEF ≤ 35%, and an implantable cardioverter-defibrillator (ICD) or cardiac resynchronization therapy defibrillator (CRT-D), death or HF hospitalization occurred in 28.5% after ablation and 44.6% with medical therapy over a median 37.8 months (HR 0.62, 95% CI 0.43–0.87). Continuous device monitoring and narrow eligibility remain central to interpretation [7,8].

Trials that did not meet their primary efficacy endpoint define the boundary of this benefit. RAFT-AF enrolled 411 patients with high-burden AF and New York Heart Association (NYHA) class II–III HF; death or HF events occurred in 23.4% with ablation-based rhythm control and 32.5% with rate control (HR 0.71, 95% CI 0.49–1.03; *p* = 0.066), and RAFT-AF did not meet its primary endpoint after early termination for futility, although LVEF, exercise capacity, and quality of life improved. AMICA likewise found similar one-year LVEF improvement after ablation and medical therapy in 140 patients with persistent AF, LVEF ≤ 35%, and an ICD or CRT-D (+8.8% vs. +7.3%; *p* = 0.36). These findings argue against using a low EF alone as an indication for ablation [9,28].

The practical signal is therefore strongest when AF burden is high, symptoms or admissions track with AF, ventricular dysfunction retains a reversible component, and procedural risk is acceptable. A 2024 phenotype-specific meta-analysis found fewer HF events with ablation in HFrEF (risk ratio [RR] 0.59, 95% CI 0.48–0.72). A later timing meta-analysis associated ablation performed early after AF diagnosis or HF decompensation with lower AF recurrence and HF hospitalization (both HR 0.63), but most pooled data were observational and definitions of “early” varied. The timing evidence favors avoiding unnecessary delay in an appropriate candidate; it does not establish a universal ablation deadline. HFmrEF after previous HFrEF or after AF-related EF decline resembles this reversible HFrEF phenotype more closely than cardiometabolic HFmrEF evolving toward HFpEF [29,30].

### 4.5. HFpEF: Symptomatic and Functional Benefit; Prognostic Evidence Remains Incomplete

In HFpEF, sinus rhythm may improve the filling-pressure reserve even when resting EF is unchanged. In a randomized trial of 31 patients with invasively confirmed HFpEF, ablation reduced peak exercise pulmonary capillary wedge pressure (PCWP) from 30.4 ± 4.2 to 25.4 ± 4.5 mmHg at six months, increased peak oxygen uptake, reduced N-terminal pro–B-type natriuretic peptide (NT-proBNP), and improved Minnesota Living with Heart Failure scores. Half of the ablation group no longer met exercise-hemodynamic criteria for HFpEF, compared with 7% receiving medical therapy. The study demonstrates a physiological and symptomatic effect but was not powered for hospitalization or mortality [19].

The original CABANA HF subgroup included 778 patients with clinical HF, most with preserved EF, and reported lower primary events, mortality, and AF recurrence with ablation; however, EF was missing in 27% and objective HFpEF criteria were not required. A 2025 CABANA analysis addressed phenotype more directly in 1763 participants. Among the 55% with a high modified H2FPEF (Heavy, Hypertensive, Atrial Fibrillation, Pulmonary Hypertension, Elder, and Filling Pressure) score, ablation was associated with a lower risk of cardiovascular hospitalization or death (HR 0.82, 95% CI 0.69–0.98), whereas no reduction was seen in those without a high HFpEF likelihood (HR 1.00, 95% CI 0.82–1.22; interaction *p* = 0.027). A sensitivity analysis in 225 patients with echocardiographic evidence of HFpEF showed a similar interaction [31,32].

A separate 2026 CABANA analysis identified probable but previously unrecognized HFpEF in 1225 participants without known HF. Ablation produced greater improvement in AF-specific symptoms and general quality of life and was associated with fewer cardiovascular hospitalizations (HR 0.78, 95% CI 0.66–0.92), but HF hospitalization itself was not reduced (HR 0.94, 95% CI 0.55–1.64). These post hoc, score-based analyses strengthen the case for recognizing HFpEF in symptomatic AF, but they do not replace a dedicated HFpEF outcome trial. Consistently, phenotype-specific randomized evidence has not shown a significant reduction in HF events in HFpEF (RR 0.93, 95% CI 0.65–1.32). Current ablation goals in HFpEF should therefore be stated as symptom relief, improved exercise hemodynamics, reduction in AF burden, and prevention of AF-related decompensation; a mortality benefit remains unproven [29,33].

### 4.6. Advanced HF, Permanent AF, and CRT

Advanced HF contains at least two clinically different populations. When long-standing AF accompanies diffuse myocardial disease, extensive atrial remodeling, or frailty, AMICA’s failure to demonstrate additional improvement in left ventricular ejection fraction supports medical rate control or a pace-and-ablate strategy rather than repeated low-yield rhythm procedures. By contrast, CASTLE-HTx enrolled 194 clinically stable patients referred to a high-volume transplant center: death, LV-assist-device implantation, or urgent transplantation occurred in 8% after ablation and 30% with medical therapy over 18 months (HR 0.24, 95% CI 0.11–0.52). Its single-center design, early stopping, and crossover restrict generalization, but the result shows that advanced HF does not uniformly preclude rhythm control when treatment is delivered within an expert HF–electrophysiology program [10,28].

Permanent AF in a cardiac resynchronization therapy (CRT) recipient is primarily a question of rate regularization and effective pacing delivery. CAAN-AF randomized 143 patients with HFrEF, permanent AF, and CRT-D to atrioventricular node ablation or medical rate control and stopped early for futility. The primary composite produced 47 events after ablation and 46 with medical therapy (incidence rate ratio [IRR] 1.16, 95% CI 0.60–2.24), with no improvement in mortality, hospitalization, exercise capacity, or quality of life. This finding should be read alongside, rather than merged with, APAF-CRT: CAAN-AF largely enrolled patients with reasonable rate control and optimized device therapy, whereas APAF-CRT addressed severely symptomatic permanent AF after HF hospitalization. Atrioventricular node ablation should therefore not be routine in every CRT-D patient with AF; its clearest role remains uncontrolled ventricular rate or failure to achieve effective biventricular pacing [34,35]. The key randomized and subgroup evidence informing rhythm-control decisions is summarized in Table 1.

### 4.7. Translating Phenotype into a Treatment Choice

**Figure 1:**
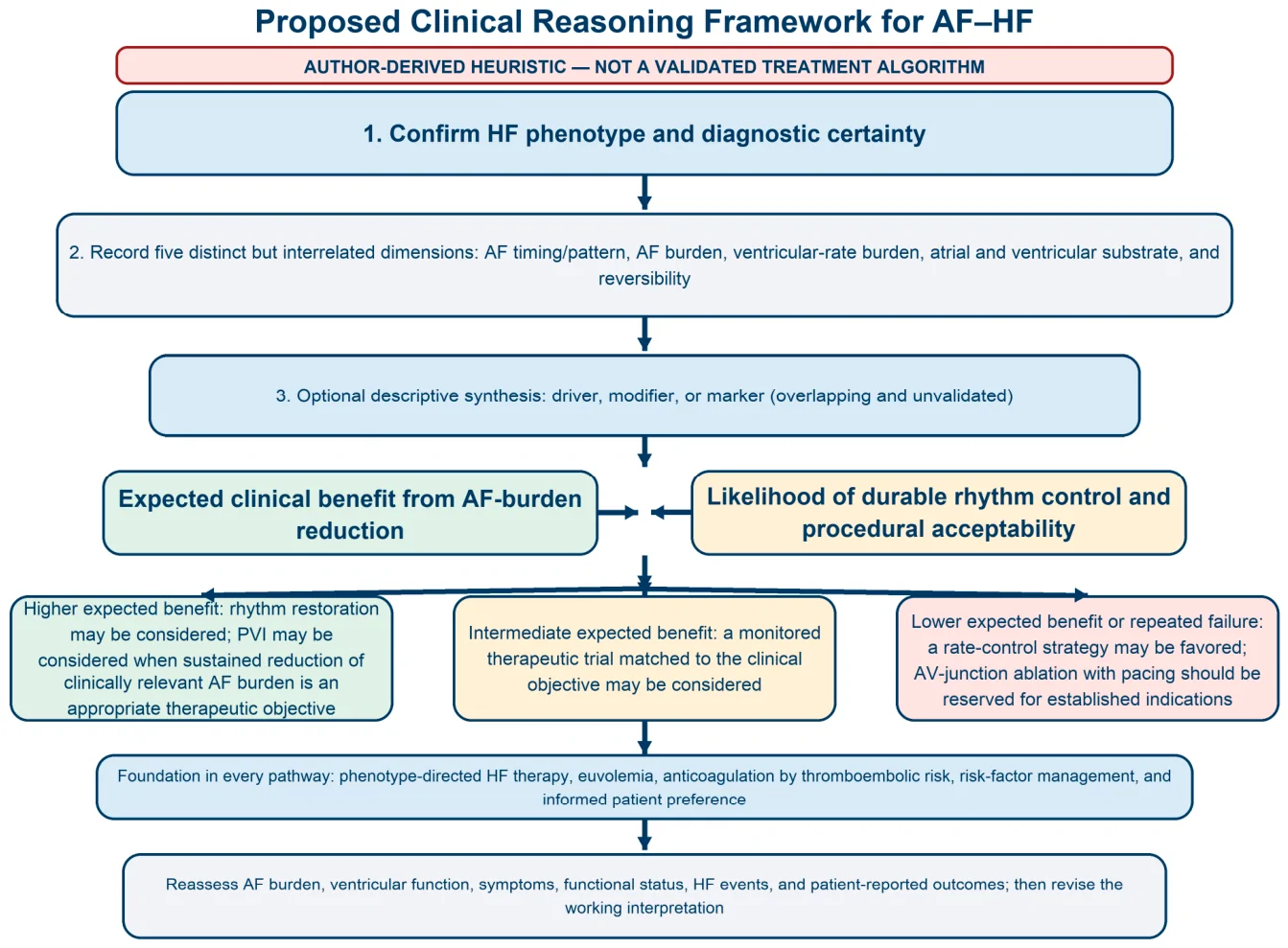
Author-derived, nonvalidated clinical reasoning framework for atrial fibrillation in heart failure; this figure is not a treatment algorithm or recommendation grade.

Table 2A–C and Figure 1 provide a proposed heuristic synthesis; they are not a validated classification, risk score, or recommendation grade. Table 2A frames testable interpretations of AF as a possible driver, modifier, or marker. Table 2B,C separate expected clinical benefit from rhythm durability and identify the evidence design supporting each scenario. Clinical options are therefore phrased as evidence-informed considerations rather than prescriptive rules.

The five clinical dimensions should be considered separately, although they are interrelated and not interchangeable. Driver–modifier–marker is an optional, overlapping author interpretation—not a validated classification or treatment score. AF, atrial fibrillation; AV, atrioventricular; HF, heart failure; LVEF, left ventricular ejection fraction; PVI, pulmonary vein isolation.

When AF causality is uncertain, HF is advanced, or repeat, hybrid, device, or pacing procedures are contemplated, treatment should be agreed upon through multidisciplinary HF and electrophysiology review with the patient. Shared decision-making should address expected symptom and prognostic benefit, rhythm durability, procedural and medication risk, monitoring burden, continued anticoagulation, access, and the possibility that electrophysiological success may not resolve patient-perceived limitation [1,26].

### 4.8. Pharmacological Rate and Rhythm Control

Drug choice follows the immediate treatment objective and hemodynamic state, not LVEF alone. Hemodynamic instability requires synchronized electrical cardioversion. In stable AF, rate control may be an initial or definitive strategy, whereas prompt rhythm restoration is prioritized when AF is temporally linked to otherwise unexplained LV dysfunction, recurrent decompensation, or ineffective CRT delivery. A lenient resting target below 110 beats/min is reasonable only while symptoms, ventricular function, exercise rate, and biventricular pacing remain acceptable; ambulatory or exercise data are more informative than one clinic pulse [1,13].

For compensated HFrEF or HFmrEF with persistent LV dysfunction, an evidence-based HF beta-blocker is used when tolerated; digoxin is an adjunct or alternative when hypotension or incomplete rate control is limiting. Renal function, interacting drugs, and serum concentration require attention; when a digoxin level is measured, a target below 1.2 ng/mL is reasonable. Verapamil and diltiazem should not be used when LVEF is ≤40%. In HFpEF with LVEF > 40%, either a beta-blocker or a non-dihydropyridine calcium-channel blocker can be selected, but excessive slowing may worsen chronotropic limitation [1,13]. RATE-AF randomized 160 older patients with permanent AF and HF symptoms to low-dose digoxin or bisoprolol. Physical quality of life at six months did not differ, but a two-class improvement in modified European Heart Rhythm Association (EHRA) symptoms occurred in 53% versus 9%, and adverse events in 25% versus 64%, respectively [41]. These results support digoxin as a rational option in selected patients, not as a universally superior first-line drug.

For long-term rhythm maintenance in HFrEF, amiodarone and, where available, dofetilide are the principal drug options. In DIAMOND-CHF, 1518 patients with symptomatic HF and severe LV dysfunction had similar mortality with dofetilide and placebo (41% versus 42%; HR 0.95, 95% CI 0.81–1.11), while HF hospitalization was reduced (RR 0.75, 95% CI 0.63–0.89). Torsade de pointes occurred in 3.3%, supporting renal- and QT interval-guided dosing with monitored inpatient initiation [42]. Amiodarone avoids this initiation pathway but cumulative thyroid, hepatic, pulmonary, ocular, and bradycardic toxicity limits long-term use. Class IC drugs and dronedarone should be avoided in HFrEF; sotalol is generally not preferred because renal clearance, QT prolongation, bradycardia, and mortality signals narrow its safety margin [1,13].

Stable HFmrEF and HFpEF permit a wider rhythm-control choice, but structural disease remains decisive. Dronedarone may be considered when LVEF is >40%, HF is stable, and AF is not accepted as permanent. In the post hoc ATHENA analysis, 534 patients had HFpEF or HFmrEF; the direction of effect favored dronedarone without heterogeneity by HF status, but individual HF-subgroup endpoints were not statistically significant [43]. Flecainide or propafenone are restricted to patients without previous myocardial infarction or significant structural heart disease, while amiodarone is reserved when safer options are unsuitable. Dronedarone should not be used in permanent AF or recently decompensated or severe HF [1,13].

Antiarrhythmic drugs should not be treated as prognostic substitutes for ablation. AF-CHF did not reduce cardiovascular mortality with drug-based rhythm control [4]. In suspected AF-mediated cardiomyopathy, medication may serve as a bridge to cardioversion or ablation. Continue treatment only while it reduces AF burden or symptoms with acceptable toxicity; recurrent high burden or intolerance should change the strategy [1,13]. Phenotype-guided pharmacological treatment considerations are summarized in Table 3.

### 4.9. Anticoagulation

Stroke prevention is independent of rate or rhythm control. The 2024 European guideline recommends oral anticoagulation at CHA_2_DS_2_-VA ≥ 2 and consideration at 1; no different threshold is defined for HFrEF, HFmrEF, or HFpEF. Direct oral anticoagulants are preferred except with mechanical valves or moderate-to-severe rheumatic mitral stenosis, with dosing based on renal function, age, weight, and interactions [1,13,44].

Cardioversion and ablation require procedure-specific anticoagulation. For elective cardioversion, the European guideline uses >24 h or uncertain duration for three weeks of therapeutic anticoagulation or transesophageal echocardiography, whereas the American guideline retains ≥48 h or an unknown duration; anticoagulation continues for at least four weeks [1,13]. Ablation is performed with uninterrupted warfarin or an uninterrupted/minimally interrupted direct oral anticoagulant. Post-ablation anticoagulation continues for at least two months in European and three months in American guidance, after which baseline thromboembolic risk—not rhythm success or HF phenotype—determines continuation [1,13,20].

Recent de-escalation trials do not establish routine anticoagulation withdrawal in AF–HF. ALONE-AF and OCEAN mainly enrolled patients with sustained rhythm control and low-to-moderate thromboembolic risk; events were rare, monitoring differed, and neither was powered for HFrEF or HFpEF [45,46,47]. Meta-analytic evidence remains largely observational [48], while left-atrial-appendage closure in OPTION reduced bleeding but adds device-related risk and is not a routine substitute for tolerated anticoagulation [49].

## 5. Catheter Ablation: Lesion Set and Energy Source

### 5.1. Pulmonary Vein Isolation as the Core Strategy

Pulmonary vein isolation (PVI) remains the required lesion set for AF ablation in HFrEF, HFmrEF, and HFpEF [20]. No randomized trial supports choosing a routine lesion set from LVEF alone. The clinical phenotype defines the value of reducing AF burden; AF pattern and duration, pulmonary vein reconnection, mapped atrial tachycardia, reproducible non-PV triggers, atrial scar, anatomy, and previous response define what is ablated.

Pulmonary vein isolation, therefore, remains the core lesion set, while additional ablation requires a mapped or reproducible mechanism rather than HF phenotype, persistent AF, atrial enlargement, or fibrosis alone [20].

### 5.2. Radiofrequency Ablation

Radiofrequency ablation creates point-by-point lesions and offers the greatest flexibility for electroanatomical mapping, complex anatomy, repeat procedures, macroreentrant atrial tachycardia, and reproducible non-PV targets. This flexibility is useful when a mapped lesion beyond PVI is expected, but it does not validate empirical lines. In CRRF-PeAF, 499 patients with persistent AF were randomized to cryoballoon or radiofrequency ablation; one-year atrial tachyarrhythmia occurred in 22.5% and 23.2%, respectively (HR 0.99, 95% CI 0.69–1.43), meeting noninferiority [50]. The multicenter randomized design and frequent electrocardiographic follow-up are strengths. However, only about 17% had HF in each group, HF-specific outcomes were not reported, adjunctive lesions were operator-selected, and monitoring was not continuous. The trial supports technical equivalence in persistent AF, not an HF-phenotype advantage for either energy. Radiofrequency retains thermal risks, including esophageal injury, steam pops, and perforation [20].

### 5.3. Cryoballoon Ablation

Cryoballoon ablation provides a standardized single-shot PVI. FIRE AND ICE showed noninferiority to radiofrequency for drug-refractory paroxysmal AF, and CRRF-PeAF extended noninferiority to persistent AF [50,51]. Cryoballoon is efficient when de novo PVI is the main objective and vein anatomy is suitable; it is less adaptable for focal mapping or complex linear lesions, and phrenic-nerve injury remains characteristic. Neither trial was designed to compare HF outcomes, and no HFrEF, HFmrEF, or HFpEF subgroup has shown prognostic superiority over radiofrequency.

### 5.4. Pulsed-Field Ablation (PFA)

Pulsed-field ablation (PFA) produces predominantly nonthermal irreversible electroporation. Collateral injury appears less frequent than with thermal energy, but tissue effects depend on waveform, dose, catheter geometry, contact, and proximity to vulnerable structures; PFA therefore represents platform-specific technologies rather than one interchangeable procedure [52].

Randomized evidence supports PFA as an effective alternative to thermal PVI, not as a uniformly superior treatment. ADVENT demonstrated noninferiority in paroxysmal AF [52,53]; PULSED AF and inspIRE were single-arm pivotal programs [54,55]; and SPHERE Per-AF and BEAT PAROX-AF reported comparable rhythm outcomes with contemporary thermal workflows [56,57]. Differences in platforms, lesion sets, monitoring, and endpoints preclude cross-trial ranking, and no study established HF-phenotype-specific prognostic superiority.

Persistent AF studies support rhythm and symptom control, but not HF hospitalization or mortality benefit. ADVANTAGE AF and AVANT GUARD used differing burden and recurrence endpoints, while PFA-SHAM excluded severe LV dysfunction, tachycardia-induced cardiomyopathy, major valve disease, important pulmonary hypertension, and marked atrial enlargement [58,59,60,61]. MANIFEST registries identified favorable but not risk-free platform-specific safety [62,63]. The 2026 HRS/EHRA statement emphasizes training, dose discipline, coronary proximity, thromboembolic protection, and surveillance [64].

HF-specific evidence remains observational. In ATHENA, one-year freedom from documented atrial arrhythmia was 73.3% in 176 patients with HF and 81.0% in those without HF, but only 40 participants had HFpEF, and EF groups were combined [65]. Implementation and patient-experience reports support feasibility, not superiority over thermal ablation for HF events or survival [66,67,68]. PFA selection should therefore reflect the planned lesion set, anatomy, platform-specific evidence, and center expertise rather than LVEF alone. Contemporary PFA platforms and their principal clinical evidence are summarized in Table 4.

### 5.5. Additional Substrate and Non-Pulmonary-Vein Ablation

The strongest older randomized evidence argues against undifferentiated lesion extension. STAR AF II found no benefit from empirical lines or complex-fractionated electrogram ablation, CAPLA found no overall benefit from empirical posterior-wall isolation, and DECAAF II did not reduce recurrence with MRI-guided fibrosis ablation and recorded more safety events [39,40,70]. In the CAPLA systolic-HF analysis, freedom from recurrence without antiarrhythmic drugs was 58.7% with PVI plus posterior-wall isolation and 61.5% with PVI alone (HR 1.02, 95% CI 0.54–1.91) [71]. These trials are persuasive against routine posterior-wall, linear, or fibrosis ablation based only on persistent AF, scar, or low EF.

Recent trials refine rather than overturn that conclusion. PROMPT-AF randomized 495 patients with persistent AF to PVI alone or PVI plus vein-of-Marshall ethanol infusion and completed mitral, roof, and cavotricuspid-isthmus lines. Single-procedure freedom from atrial arrhythmia without antiarrhythmic drugs was 70.7% versus 61.5% (HR 0.73, 95% CI 0.54–0.99; *p* = 0.045) [72]. Its multicenter randomized design and verification of line block are strengths. However, mean LVEF was about 61%, only 53 patients had HF and 34 had LVEF < 50%, continuous monitoring was not used, AF burden and quality-of-life changes did not differ, and pericarditis or effusion occurred in seven intervention patients versus none with PVI alone. Because the intervention was a package, the effective component cannot be isolated.

The smaller single-center Marshall-PLAN trial reported one-year freedom from atrial arrhythmia in 86.4% with the combined Marshall/three-line strategy and 66.1% with PVI alone (*p* = 0.012) [73]. The lesion set was completed in 88%, but the procedure and fluoroscopy times were longer; only 118 patients were analyzed, follow-up was 12 months, and monitoring was intermittent. Together with VENUS [74], these data make Marshall-facilitated line completion a credible option for selected persistent AF, not a default HF lesion set.

The 2026 EXT-AF&HF trial is the most directly relevant new evidence. Three hundred patients with persistent AF and HF were randomized to PVI plus anatomic ablation, electrogram-guided ablation, or both. At 36 months, cardiovascular death or an HF-related admission or urgent visit occurred in 36%, 29%, and 17%, while sinus rhythm was maintained in 44%, 53%, and 62%, respectively [36]. Long follow-up, randomization, an HF-specific population, and clinical as well as rhythm endpoints are important strengths. Interpretation remains cautious: there was no PVI-only arm, electrogram targets are mapping- and operator-dependent, the HFpEF category included LVEF 41–50%, and subgroup trends do not prove equal benefit in current HFrEF, HFmrEF, and HFpEF definitions. External replication is needed before extensive electrogram-anatomic ablation becomes routine.

### 5.6. Repeat, Surgical, and Hybrid Ablation

After recurrence, pulmonary-vein reconnection is assessed first and re-isolation remains the best-established repeat strategy. A repeat procedure has the clearest value when the first burden reduction improved EF, symptoms, exercise tolerance, or HF stability. Surgical or hybrid ablation is reserved for selected patients after failed catheter procedures, during concomitant cardiac surgery, or with long-standing persistent AF and major remodeling, within an experienced multidisciplinary team [20]. HALT-AF is designed to randomize 120 patients with nonparoxysmal AF, LVEF < 50%, and at least moderate left-atrial dilatation to lesion-matched convergent hybrid or endocardial ablation [75]. Its lesion-matched HF-specific design addresses an important gap, but it is a protocol; until outcome data are available, it cannot support routine hybrid ablation.

### 5.7. Atrioventricular Node Ablation and Pacing

Atrioventricular junction ablation provides complete rate regularization when rhythm control is unsuccessful or inappropriate. The relevant profiles are permanent AF with refractory rapid or irregular rates, recurrent HF admissions despite medication, or inadequate biventricular pacing during AF. In APAF-CRT, 133 patients with severely symptomatic permanent AF, QRS ≤ 110 ms, and a prior HF hospitalization were randomized to atrioventricular junction ablation plus CRT or drug rate control. Mortality was 11% versus 29% (HR 0.26, 95% CI 0.10–0.65), and death or HF hospitalization was 29% versus 51% (HR 0.40, 95% CI 0.22–0.73) over a median of 29 months [34].

Pacing modality is chosen before atrioventricular junction ablation. CRT remains the evidence-based option when LVEF is reduced, conventional CRT indications are present, or effective biventricular pacing cannot be achieved during AF. His-bundle pacing and left-bundle-branch-area pacing offer physiologic activation for narrow-QRS patients who will become pacing-dependent, but randomized outcome evidence is less mature than for CRT [38]. Isolated right-ventricular pacing is avoided when a high pacing burden creates a meaningful risk of pacing-induced cardiomyopathy. Pace-and-ablate does not restore atrial contraction or remove the need for anticoagulation.

### 5.8. Post-Ablation Follow-Up and Patient Expectations

Post-ablation care should clarify that procedural success usually refers to reduction in AF burden or recurrence rather than complete resolution of the disease. Matteucci et al. emphasize that electrophysiological success and patient-perceived recovery may diverge, with residual symptoms, impaired quality of life, anxiety, treatment concerns, or dissatisfaction despite apparently effective rhythm control [26]. Because this contribution is an editorial, it supports the need for patient-centered follow-up rather than a comparative treatment-effect estimate. Rhythm monitoring and validated quality-of-life assessment should therefore match the original treatment objective. Anticoagulation after the mandatory post-procedure period remains based on thromboembolic risk [1,13,45,46,47,48,49], while HF disease-modifying therapy and risk-factor management generally continue unless separate evidence supports adjustment [16,17,20,21,22]. Follow-up may include AF burden, ventricular function, congestion, functional capacity, medication adverse effects, patient-reported outcomes, and the possible value of repeat intervention [1,20,26].

## 6. Heart Failure Optimization and Prevention of AF Recurrence

Rate or rhythm control should be delivered on a background of optimized HF therapy and euvolemia. In HFrEF, guideline-directed management generally includes an angiotensin receptor–neprilysin inhibitor, angiotensin-converting enzyme inhibitor, or angiotensin receptor blocker; an evidence-based beta-blocker; a mineralocorticoid receptor antagonist; and a sodium–glucose cotransporter 2 inhibitor, initiated and titrated as tolerated. Diuretics are used to control congestion, while renal function, potassium, iron status, ischemia, valve disease, and device indications require parallel assessment [16,17,37]. Restoration of sinus rhythm does not replace disease-modifying HF therapy. In HF with improved ejection fraction or recovered AF-mediated cardiomyopathy, routine treatment withdrawal is not supported; WITHDRAW-AF and DEFINITION-AF permit only carefully selected, closely monitored de-escalation [21,22].

In HFmrEF and HFpEF, sodium–glucose cotransporter 2 inhibitors are recommended to reduce HF hospitalization or cardiovascular death, while diuretics treat congestion and other therapies are individualized according to previous HFrEF, blood pressure, renal function, and specific indications [16,17]. Hypertension, obesity, diabetes, chronic kidney disease, ischemia, valve disease, and sleep apnea should be addressed because they influence filling pressure, atrial remodeling, symptom attribution, and rhythm durability. HF therapies should be prescribed for their established HF indications rather than as surrogate antiarrhythmic drugs. Although a trial-level meta-analysis suggested a modest reduction in incident AF with sodium–glucose cotransporter 2 inhibition, DARE-AF randomized 200 patients without HF, diabetes, or chronic kidney disease and did not reduce three-month AF burden or atrial-arrhythmia recurrence [76,77].

Structured management of weight, blood pressure, sleep apnea, physical inactivity, alcohol exposure, and diabetes is part of the rhythm-control strategy rather than ancillary counseling. In ARREST-AF, structured risk-factor and weight management improved ablation outcomes among patients with elevated body mass index and additional cardiometabolic risk [78]. The relevance is greatest in HFpEF and obesity-related atrial myopathy, but implementation depends on sustained follow-up and access to multidisciplinary care.

## 7. Ongoing Trials and Future Evidence

The major unresolved question is no longer whether AF burden can be reduced, but which HF phenotypes derive durable clinical benefit beyond rhythm and symptom endpoints. Several active studies address this gap with different populations and control strategies (Table 5). CRAAFT-HF is the largest reduced-EF strategy trial and permits radiofrequency, cryoballoon, or PFA at clinician discretion [79]. CABA-HFPEF, STABLE-SR IV, CAPHF-AF, CABANA-RAFT HF, and CASTLE-HFpEF focus on HFmrEF/HFpEF but differ in diagnostic entry criteria, monitoring, comparator therapy, and primary outcome [80,81,82,83,84]. These design differences will matter when the results are compared.

PFA-specific HF evidence also remains immature. CASTLE-HFpEF plans a PFA-based ablation strategy with continuous rhythm monitoring, whereas a smaller persistent-AF/HFpEF study evaluates a circular PFA platform against antiarrhythmic-drug therapy [84,85]. HALT-AF separately tests lesion-matched convergent hybrid versus endocardial ablation in nonparoxysmal AF with LVEF < 50% [75]. Until these studies are reported, the ongoing status should not be presented as efficacy evidence, and extrapolation among PFA platforms or from broad AF cohorts to HFrEF or HFpEF should remain cautious.

## 8. Discussion

A central interpretation of this review is that left ventricular ejection fraction alone may not define the clinical role of AF within the HF syndrome. Treatment selection may be better informed by five distinct observations—AF timing/pattern, quantified AF burden, ventricular-rate burden, atrial and ventricular substrate, and reversibility—because these dimensions are related but not interchangeable. The atrial-cardiomyopathy model discussed by Pierucci et al. offers a plausible biological context linking systemic risk factors, HF, AF persistence, and thromboembolic risk, but it does not provide a validated ablation-selection score [14]. The driver–modifier–marker terminology is therefore retained only as an author-derived clinical interpretation that may be strengthened when verified burden reduction is followed by ventricular, hemodynamic, functional, or symptomatic improvement [2,15].

Apparent disagreement among rhythm-control trials is better explained by differences in treatment context than by a simple historical transition from ineffective drugs to effective ablation. AF-CHF tested predominantly drug-based rhythm control in established systolic HF, whereas EAST-AFNET 4 tested an early strategy and CAMERA-MRI tested reversibility despite adequate resting rate control. RATE-AF and DIAMOND-CHF further show that a drug should be judged against a defined clinical objective and toxicity profile rather than assigned a universal class effect [4,6,11,41,42]. Timing, achieved burden reduction, monitoring intensity, and competing myocardial disease are consequently central to interpretation.

The same considerations explain why the evidence threshold differs across HF phenotypes. Selected HFrEF populations provide the strongest randomized evidence for reducing HF events, but device-based monitoring, close follow-up, and narrow eligibility limit generalization. Trials that did not meet their primary endpoint show why low EF alone is insufficient. In HFpEF, the established signal is different: symptom relief, improved exercise hemodynamics, quality of life, and lower AF burden are plausible and clinically important, but they do not yet establish phenotype-specific survival benefit. Advanced HF likewise should not be treated as a uniform futility category; the relevant question is whether AF remains a modifiable component within an expert HF–electrophysiology program [7,8,9,10,19,28,29,32,33].

Energy-source selection and lesion-set design answer different questions. PVI remains the procedural foundation, while radiofrequency, cryoballoon, and PFA alter workflow, mapping flexibility, and the profile of collateral-tissue injury. Contemporary PFA trials and registries support efficient rhythm control and a favorable safety profile, but the results are platform-specific and randomized HF outcomes are absent. A shorter procedure or lower incidence of one complication cannot be translated automatically into better HF survival. Similarly, the neutral results of empirical posterior-wall or fibrosis ablation argue against lesion extension based on EF or persistent AF alone, whereas newer positive studies support the selective use of reproducible, mechanistically coherent targets. No single study yet justifies a routine HF-specific energy or adjunctive-lesion algorithm [36,39,40,52,53,54,55,56,57,58,59,60,61,62,63,64,65,66,67,68,69,70,71,72,73].

Treatment success may be considered multidimensional. AF burden can be more informative than a single 30 s recurrence when ventricular recovery, CRT delivery, or HF events are the clinical objectives [24,25], but electrical control and patient-perceived recovery may diverge. The patient-perspective editorial by Matteucci et al. supports pairing rhythm data with symptoms, functional capacity, anxiety about recurrence, treatment burden, and validated quality-of-life measures [26]. Stroke prevention remains a separate risk-based decision; current de-escalation trials do not support routine anticoagulation withdrawal in higher-risk AF–HF populations [1,13,45,46,47,48,49].

Clinical applicability also depends on access that is not captured by ejection fraction or rhythm endpoints. In a large cross-sectional database, women, racial and ethnic minority groups, and patients insured through Medicaid had lower rates of AF ablation, indicating that use of the procedure is not determined by clinical phenotype alone [86]. Socioeconomic constraints and the burden of repeated visits may also affect whether a technically appropriate rhythm-control strategy is feasible; these factors should be addressed during shared decision-making.

This narrative review has limitations. Study selection was purposive; duplicate independent screening, protocol registration, formal risk-of-bias assessment, quantitative synthesis, and complete cross-source record-level deduplication were not performed. The design labels used in the manuscript improve transparency but are not a formal GRADE certainty assessment. HF definitions, rhythm monitoring, burden thresholds, ablation protocols, antiarrhythmic use, and follow-up varied substantially. HFpEF evidence often relied on small randomized studies or probability-based post hoc analyses, while PFA findings remain platform-specific and HF outcome data are predominantly observational. Registry entries cannot be interpreted as efficacy evidence. The driver–modifier–marker framework is unvalidated, and several 2025–2026 findings require independent replication.

## 9. Conclusions

Phenotype-guided management integrates the clinical role of AF with the expected benefit and durability of AF-burden reduction. The driver–modifier–marker terminology is an unvalidated descriptive framework and must not be used as a stand-alone treatment score. Suspected AF-mediated cardiomyopathy should prompt consideration of early rhythm restoration, whereas selected patients with HFrEF- and AF-related symptoms or hospitalizations have the strongest evidence for PVI-based ablation. In HFpEF, established objectives are improvements in symptoms, functional capacity, exercise hemodynamics, and AF burden rather than mortality reduction. PVI remains the procedural core; adjunctive ablation should be mechanism-based. Radiofrequency, cryoballoon, and PFA should be selected according to anatomy, procedural objective, platform-specific evidence, safety, and center expertise rather than HF phenotype. Rate regularization and pacing remain appropriate when durable rhythm control is unlikely or ineffective. Ongoing trials will clarify hard outcomes in HFmrEF/HFpEF and the role of PFA, but their registry status is not evidence of benefit. Phenotype-directed HF therapy, euvolemia, stroke prevention, risk-factor management, multidisciplinary review, and informed patient preference are integral to every strategy. Apparent rhythm success does not by itself justify withdrawal of HF therapy or anticoagulation.

## Figures and Tables

**Table 1:** Key randomized and prespecified or post hoc evidence informing rhythm-control decisions in atrial fibrillation and heart failure.

Trial	Evidence Design	Phenotype, Sample, and Follow-Up	Main Result	Interpretation/Limitation
AF-CHF [4]	Direct randomized strategy trial; drug-dominant rhythm control	HFrEF, LVEF ≤ 35%; *n* = 1376; 37 months	Cardiovascular death: 27% rhythm vs. 25% rate; HR 1.06; *p* = 0.59	Frequent AF recurrence and substantial amiodarone exposure; does not test early ablation.
EAST-AFNET 4 HF [6]	Prespecified HF subgroup of a randomized trial	Recent AF plus HF; *n* = 798; LVEF available in 785; 5.1 years	Primary composite: HR 0.74 (95% CI 0.56–0.97)	Supports early rhythm control as a strategy; ablation was only one component.
AATAC [7]	Direct randomized trial in selected device-treated HFrEF	Persistent AF, LVEF < 40%, ICD/CRT-D; *n* = 203; 24 months	AF-free: 70% vs. 34%; hospitalization: 31% vs. 57%; mortality: 8% vs. 18%	Ablation outperformed amiodarone; selected population limits generalizability.
CAMERA-MRI [11]	Direct small mechanistic randomized trial	Persistent AF, unexplained LVEF ≤ 45%; *n* = 68; 6 months	LVEF: +18% vs. +4.4%; normalization: 58% vs. 9%	Strong reversibility signal; small sample and imaging endpoint.
CASTLE-AF [8]	Direct randomized trial in continuously monitored HFrEF	Symptomatic AF, LVEF ≤ 35%, ICD/CRT-D; *n* = 363; 37.8 months	Death/HF admission: 28.5% vs. 44.6%; HR 0.62 (95% CI 0.43–0.87)	Hard-outcome benefit in a narrowly defined, device-monitored cohort.
AMICA [28]	Direct randomized trial; neutral primary result	Persistent AF, LVEF ≤ 35%, ICD/CRT-D; *n* = 140; 12 months	LVEF: +8.8% vs. +7.3%; *p* = 0.36	No incremental LVEF benefit; stopped early for futility.
RAFT-AF [9]	Direct randomized strategy trial; stopped early	High-burden AF, NYHA II–III HF; *n* = 411; ≥24 months	Death/HF events: 23.4% vs. 32.5%; HR 0.71 (95% CI 0.49–1.03); *p* = 0.066	Primary endpoint not met; functional endpoints improved.
CABANA-HF [31]	Post hoc clinical-HF subgroup of a randomized trial	Clinical HF, predominantly preserved EF; *n* = 778; 48.5 months	Primary composite: HR 0.64; mortality: HR 0.57	EF missing in 27%; objective HFpEF criteria were not required.
HFpEF randomized trial [19]	Direct small mechanistic randomized trial	Invasively confirmed HFpEF; *n* = 31; 6 months	Peak exercise PCWP fell from 30.4 to 25.4 mmHg after ablation; *p* < 0.01	Physiological and symptom benefit; not powered for hospitalization or mortality.
CASTLE-HTx [10]	Direct single-center randomized trial in highly selected advanced HF	Stable transplant-evaluation HF; *n* = 194; 18 months	Death/LVAD/urgent transplant: 8% vs. 30%; HR 0.24	Open label, early stopping, crossover, and expert-center setting restrict generalization.
CABANA-HFpEF [32]	Post hoc probability-based analysis	Modified H2FPEF analysis; *n* = 1763; 48.5 months	High HFpEF likelihood: cardiovascular admission/death HR 0.82; interaction *p* = 0.027	Hypothesis-generating phenotype analysis; sensitivity cohort *n* = 225.
WITHDRAW-AF/DEFINITION-AF [21,22]	Small pilot withdrawal studies	Recovered AF-mediated cardiomyopathy; *n* = 60 and *n* = 50; 6–12 months	LVEF ≥50% maintained: 90% vs. 100%; HF deterioration: 13% withdrawal vs. 0% continuation	Hypothesis-generating; does not support routine HF-therapy withdrawal.
CAAN-AF [35]	Direct randomized trial; stopped early for futility	Permanent AF, HFrEF, CRT-D; *n* = 143; 24 months	Death/nonfatal HF events: IRR 1.16 (95% CI 0.60–2.24)	Baseline rate control and CRT delivery were generally adequate.
EXT-AF&HF [36]	Direct HF-specific randomized trial; no PVI-only arm	Persistent AF plus HF across EF; *n* = 300; 36 months	CV death/HF admission or urgent visit: 17% extensive vs. 29% electrogram-guided vs. 36% anatomic-guided	Mapping was operator-dependent; phenotype subgroups and lesion components need replication.

**Table 2:** (**A**) Proposed, nonvalidated descriptive framework for interpreting AF as a possible driver, modifier, or marker in heart failure. (**B**) Evidence-informed treatment considerations for foundational management and AF–HF scenarios with higher or intermediate expected benefit from AF-burden reduction. (**C**) Evidence-informed treatment considerations for advanced substrate, permanent AF, and recurrence after prior pulmonary vein isolation.

(**A**)				
Provisional clinical role	Supporting findings	Weakening findings	Questions to test the interpretation	Findings that may shift the interpretation
Driver-predominant: suspected AF-mediated cardiomyopathy	Support: AF precedes otherwise unexplained LV dysfunction; verified burden is high; competing disease and scar are limited; prior sinus rhythm improved function.	Weaken: dysfunction clearly predates AF or persists despite major burden reduction [11,27].	Record the AF–HF timeline; quantify burden and ventricular-rate exposure; assess atrial and ventricular substrate, valves, and ischemia; test reversibility rather than relying on resting rate alone [12,15].	Recovery after verified burden reduction supports a causal role. Absent recovery shifts weight toward modifier or marker; HF surveillance continues after recovery [21,22].
Modifier-predominant: AF aggravates established HF	Support: HF predates AF, but congestion, functional mitral regurgitation, exercise limitation, admissions, or ineffective CRT delivery track with AF.	Weaken: symptoms and HF events do not vary with rhythm or burden [2,37].	Relate episodes and ventricular rates to symptoms, admissions, biomarkers, exercise findings, and device data; define the clinical objective before selecting rhythm or rate control [1].	Improved symptoms, hemodynamics, HF stability, or pacing delivery supports a modifier role. Persistent deterioration despite major burden reduction increases the weight of underlying HF [2].
Marker-predominant: AF accompanies advanced substrate	Support: long-standing AF; severe biatrial enlargement or fibrosis; pulmonary hypertension, right-ventricular failure, major valve disease, frailty, or repeated rhythm-control failure.	Weaken: recent AF, limited scar, and reproducible response to sinus rhythm [20,28].	Define atrial and ventricular substrate, frailty, procedural risk, prior rhythm response, and the patient’s goal; reserve another rhythm attempt for a clear, achievable benefit [20].	A reproducible response to cardioversion identifies a remaining modifiable component. No clinical gain despite burden reduction favors symptom-directed rate control or pacing when indicated [10,35].
(**B**)				
Scenario (provisional AF role)	Expected benefit/evidence	Rhythm durability	Evidence-informed options	Reassessment
Foundational management in all AF–HF phenotypes	Optimize congestion, reversible precipitants, phenotype-directed HF therapy, and stroke prevention before attributing symptoms to AF [1,16,17]. Guideline/consensus basis.	Reassess after stabilization; HF therapy may change LVEF, filling pressure, symptoms, and AF burden.	Joint HF–electrophysiology review may help when causality or procedure choice is uncertain; include frailty, access, treatment burden, and preference [1,20,26].	Track congestion, renal function, treatment tolerance, AF burden, function, and patient-reported outcomes.
Suspected AF-mediated cardiomyopathy; possible driver	Benefit may be greatest when AF precedes unexplained LV dysfunction and verified burden is high; the objective is ventricular recovery [11]. Small randomized/pilot evidence.	More favorable with recent AF, smaller atria, limited scar, and prior sinus-rhythm response; less favorable with extensive fibrosis or valve disease.	Cardioversion can test reversibility; recurrent high burden may support PVI-based ablation after discussion of drug and procedural risks.	Reassess AF burden, LVEF, symptoms, and HF stability after 3–6 months; recovery does not justify routine HF-therapy withdrawal [21,22].
Recent AF with HF across LVEF; possible driver or modifier	Early rhythm control reduced events in a prespecified HF subgroup [6]; this was not an ablation-only trial.	Often higher earlier in disease; remodeling, longer AF duration, and untreated risk factors reduce durability [23].	Cardioversion, an appropriate antiarrhythmic drug, or PVI may be selected according to AF burden, symptoms, HF events, contraindications, and preference—not LVEF alone.	Track AF burden, symptoms, LVEF, and HF events; absent benefit despite burden reduction requires reassessment.
Selected HFrEF or potentially reversible HFmrEF; possible driver or modifier	Selected randomized HFrEF trials support fewer HF admissions and improved function, with a survival signal in narrow populations [7,8].	Substrate-dependent; scar, atrial enlargement, frailty, renal dysfunction, and long-standing AF reduce durability [9,28].	After HF optimization, PVI may be considered; low LVEF alone does not justify empirical posterior-wall, linear, or fibrosis ablation.	Assess AF burden, LVEF, function, symptoms, and HF admissions; no clinical gain argues against serial procedures.
HFpEF with symptomatic or hemodynamic AF; possible modifier	Evidence supports symptom, exercise-hemodynamic, quality-of-life, and AF-burden improvement; mortality benefit remains unproven [19,32,33]. Small direct/post hoc evidence.	Variable; earlier AF and less remodeling may favor durability, whereas advanced atrial cardiomyopathy and uncontrolled risk factors may reduce it.	PVI may be considered in appropriate patients. Select energy by anatomy, workflow, platform-specific safety, and expertise; no HFpEF-specific prognostic superiority is established.	Use AF burden, quality of life, exercise capacity, congestion, and decompensation; persistent symptoms require assessment of non-AF mechanisms.
(**C**)				
Scenario (provisional AF role)	Expected benefit/evidence	Rhythm durability	Evidence-informed options	Reassessment
Advanced HF with diffuse atrial and ventricular disease; possible marker	Benefit is uncertain and most plausible when a reversible AF component can be demonstrated; selected trials should not be generalized to all advanced HF [10,28].	Often low with long-standing AF, extensive fibrosis, valve disease, pulmonary hypertension, right-ventricular dysfunction, or frailty.	Prioritize HF optimization and symptom-directed rate control. PVI may be considered only after multidisciplinary review; avoid empirical lesion extension.	Reassess frailty, scar, atrial size, prior rhythm response, and device/transplant trajectory; no measurable gain argues against further procedures.
Permanent AF with uncontrolled rate or ineffective CRT delivery	The objective is rate regularization and effective pacing, not recovery of atrial contraction; trials studied clinically different populations.	Sinus-rhythm durability is no longer the objective; success depends on rate control and pacing delivery.	Optimize medication and CRT programming first. AV-junction ablation with appropriate pacing may be considered for uncontrolled rate, recurrent HF admission, or ineffective pacing—not routine in well-controlled CRT [34,35,38].	Monitor ambulatory rate, effective biventricular pacing, symptoms, and HF admissions; no clinical gain requires substrate reassessment.
Clinically important recurrent AF after prior PVI	Repeat intervention is most defensible after prior measurable improvement in AF burden, LVEF, symptoms, exercise tolerance, or HF stability. Consensus/mechanism-based evidence.	Pulmonary-vein reconnection or mapped atrial tachycardia is more actionable than diffuse atrial disease without a reproducible target.	Re-isolate reconnected veins and treat mapped tachycardia or reproducible non-PV triggers; empirical posterior-wall, linear, or fibrosis ablation remains unsupported [20,39,40].	Define success by AF burden and clinical benefit; without a treatable mechanism, favor medication, rate control, pacing when indicated, or symptom-directed care.

**Table 3:** Phenotype-guided use of rate-control and antiarrhythmic drugs in atrial fibrillation and heart failure.

Phenotype and Treatment Objective	Rate-Control Approach	Rhythm-Control Approach	Reassessment and Main Cautions
Suspected AF-mediated cardiomyopathy or recent AF-related LVEF decline	Use rate control for stabilization, not as proof that AF is benign. A beta-blocker is used when compensated; digoxin is an option when hypotension limits therapy.	Prompt cardioversion tests reversibility. If LVEF is ≤40%, amiodarone or, where available, dofetilide may support rhythm restoration; recurrent high burden supports early PVI-based ablation.	Document AF burden and serial LVEF. Move away from repeated drug escalation when rhythm is not durable or toxicity develops [1,13,42]
Compensated HFrEF (LVEF ≤ 40%)	Use an evidence-based HF beta-blocker if tolerated; add or substitute digoxin when blood pressure or rate control is limiting. Avoid verapamil and diltiazem.	Amiodarone or, where available, dofetilide are the principal drug options. Avoid class IC drugs and dronedarone; sotalol is generally not preferred.	Monitor blood pressure, renal function, QT interval, digoxin exposure, and amiodarone organ toxicity. Dofetilide requires monitored inpatient initiation [1,13,42]
HFmrEF or HF with improved ejection fraction	Use the previous as well as the current LVEF. A patient with recent HFrEF or incomplete recovery is treated with the same rate-control cautions as HFrEF.	When LVEF is >40% and HF is stable, dronedarone may be considered; amiodarone is reserved for selected patients. Class IC drugs require absence of prior myocardial infarction or significant structural disease.	Reassess if LVEF or HF status changes. Apparent recovery does not automatically remove drug-related risk [1,13,43]
HFpEF (LVEF ≥ 50%) with symptomatic or hemodynamic AF	A beta-blocker or, when LVEF is >40%, diltiazem or verapamil may be used. Low-dose digoxin is reasonable in selected older patients; avoid excessive slowing when chronotropic limitation is present.	Dronedarone is an option when HF is stable. Flecainide or propafenone requires absence of prior MI and significant structural disease; reserve amiodarone when safer options are unsuitable.	Judge benefit by symptoms, exercise capacity, congestion, and AF burden. Monitor renal function, QT interval, and bradycardia [1,13,41,43]
Acute decompensated or hemodynamically unstable HF	Instability requires synchronized electrical cardioversion. If urgent pharmacological rate control is needed with severe LV dysfunction, digoxin or intravenous amiodarone may be used; avoid intravenous verapamil or diltiazem and aggressive beta-blockade.	Electrical cardioversion is preferred for instability. Intravenous amiodarone may assist rate or rhythm control when other agents are unsuitable, with attention to embolic risk.	After stabilization, reassess the HF phenotype, AF timing, anticoagulation, and the need for a durable rhythm strategy [1,13]
AF accepted as permanent, or permanent AF with ineffective CRT delivery	Tailor beta-blocker and/or digoxin to symptoms and ventricular rate. In CRT recipients, effective biventricular pacing is the target rather than one clinic heart rate.	Do not use chronic antiarrhythmic medication solely for rate control. Consider atrioventricular-junction ablation with an appropriate pacing strategy when rate or CRT delivery remains inadequate.	Track symptoms, HF admissions, rate profile, and effective biventricular pacing; adequate rate without clinical gain should prompt reassessment of the HF substrate [1,13,34,35]

**Table 4:** Contemporary pulsed-field ablation platforms and principal clinical evidence.

Platform Architecture	Evidence Type and Representative Studies	What Is Supported	What Remains Uncertain in HF
Pentaspline single-shot PFA	Randomized thermal comparison plus single-arm/registry studies: ADVENT, ADVENT-LTO, BEAT PAROX-AF, ADVANTAGE AF, AVANT GUARD, SINGLE SHOT CHAMPION [52,53,57,58,60,69]	Efficient wide-area PVI; noninferior rhythm control in broad AF populations; the largest post-market safety experience.	Platforms and endpoints are not interchangeable; no randomized HF hard-outcome evidence.
Circular multielectrode PFA	Single-arm pivotal evidence: PULSED AF [54]	Standardized single-shot workflow with prospective paroxysmal- and persistent-AF cohorts.	Single-arm effectiveness; no HF-specific randomized trial or prognostic endpoint.
Variable-loop, mapping-integrated PFA	Single-arm pivotal evidence: inspIRE [55]	Real-time mapping integration and adaptable loop geometry.	Application count and workflow influence outcome; HF-specific evidence is lacking.
Lattice-tip dual-energy PFA/RF	Randomized platform/workflow comparison: SPHERE Per-AF [56]	One catheter for mapping and PFA or RF; noninferior rhythm outcome with shorter procedures.	Not a pure energy comparison; intermittent monitoring and no HF-specific endpoint.
Focal or large-tip PFA systems	Early feasibility and scientific-statement evidence [64]	Potential flexibility for focal targets or tailored lesion delivery.	Early platform-specific data; lesion depth, coronary proximity, cerebral safety, and durability require validation.

AFEQT, atrial fibrillation effect on quality of life; AF, atrial fibrillation; HF, heart failure; PFA, pulsed-field ablation; PVI, pulmonary vein isolation; RF, radiofrequency.

**Table 5:** Selected ongoing trials addressing atrial fibrillation ablation in heart failure (registry status checked 30 July 2026).

Trial/Registry	Population and Design	Intervention/Comparator	Primary Objective and Expected Completion
HALT-AF; NCT05411614 [75]	*n* = 120; nonparoxysmal AF, LVEF < 50%, and at least moderate left-atrial dilation; randomized.	Convergent hybrid versus lesion-matched endocardial PVI plus posterior-wall isolation.	Freedom from atrial arrhythmia off antiarrhythmic drugs; protocol-stage evidence.
CRAAFT-HF; NCT06505798 [79]	Target *n* = 1200; AF with LVEF < 50%; randomized, recruiting.	Catheter ablation (RF, cryoballoon, or PFA) plus medical therapy versus medical therapy.	All-cause death or urgent cardiovascular hospitalization; estimated completion 2031.
CABA-HFPEF; NCT05508256 [80]	Target *n* = 1548; symptomatic HFmrEF/HFpEF and AF; randomized.	Early catheter ablation versus usual medical care without planned ablation.	Cardiovascular death, stroke, and total unplanned cardiovascular hospitalization; estimated completion 2027.
STABLE-SR IV; NCT06125925 [81]	Target *n* = 436; AF with HFpEF; randomized, outcome-assessor blinded.	Radiofrequency ablation versus medical therapy.	Cardiovascular death or worsening-HF hospitalization/urgent visit; follow-up to 36 months.
CAPHF-AF; NCT06740539 [82]	Target *n* = 304; recent paroxysmal or persistent AF with HFpEF; randomized.	Catheter ablation versus HF therapy plus rate control.	All-cause death or rehospitalization for worsening HF at 24 months; estimated completion 2027.
CABANA-RAFT HF pilot; NCT07272902 [83]	Target *n* = 84; AF with HFmrEF/HFpEF; randomized, recruiting.	Ablation-based rhythm control versus rate-control medication.	Pilot feasibility, quality of life, AF burden, and clinical outcomes; estimated completion 2027.
CASTLE-HFpEF; NCT07254455 [84]	Target *n* = 900; recent AF, LVEF > 40%, objective HF, and continuous monitoring; not yet recruiting at the status date.	PFA-based ablation versus conventional medical AF care.	Hierarchical death, stroke/TIA, HF hospitalization, and NT-proBNP endpoint; estimated completion 2031.
Persistent AF–HFpEF PFA study; NCT07077811 [85]	Target *n* = 158; persistent AF with HFpEF; prospective randomized study, enrolling by invitation.	Circular multielectrode PFA versus class I/III antiarrhythmic-drug therapy.	Change in AFEQT at 12 months, with rhythm and HF secondary endpoints; estimated completion 2028.

AF, atrial fibrillation; AFEQT, atrial fibrillation effect on quality of life; HF, heart failure; HFmrEF, heart failure with mildly reduced ejection fraction; HFpEF, heart failure with preserved ejection fraction; LVEF, left ventricular ejection fraction; NT-proBNP, N-terminal pro-B-type natriuretic peptide; PFA, pulsed-field ablation; PVI, pulmonary vein isolation; RF, radiofrequency; TIA, transient ischemic attack. Registry status and projected dates may change; registry entries do not establish efficacy.

## Data Availability

Dated search exports, query files, API metadata, and PubMed–OpenAlex matching outputs are provided in the separate Supplementary Data S1 archive. No patient-level or original clinical data were generated or analyzed.

## Supplementary Materials

The following supporting information can be downloaded at: https://www.mdpi.com/article/10.3390/jcm15176519/s1, Table S1: Exact database strategies; Table S2: Dated search yields and deduplication; Table S3: Narrative evidence scope and handling; Data S1 (separate ZIP archive): Search_Archive_2026-07-30. dated PubMed and OpenAlex verification exports, executable query files, API metadata, and PubMed–OpenAlex matching outputs.
