# Supplementary material for: Phenotype-Guided Management of Atrial Fibrillation in Heart Failure: From Rate Control to Catheter Ablation
Source: J Clin Med. 2026 Aug 23;15(17):6519. doi: 10.3390/jcm15176519 (PMC13566356; doi:10.3390/jcm15176519)
Supplement: Supplementary file 1 [file jcm-15-06519-s001.zip › jcm-4504387-supplementary.pdf]

SUPPLEMENTARY SEARCH STRATEGY

Phenotype-Guided Management of Atrial Fibrillation in Heart Failure: From Rate Control to Catheter Ablation

Search through: 29 July 2026; targeted update: 30 July 2026

This supplement records the exact database strategies and dated source yields used to inform a critical narrative review. The figures describe retrieval and deduplication rather than a formal study-selection flow. Study selection was purposive; duplicate independent screening, protocol registration, a PRISMA-style selection process, quantitative pooling, and formal risk-of-bias or GRADE assessment were not undertaken. Dated machine-readable verification exports, query files, OpenAlex API parameters, and PubMed–OpenAlex matching outputs are provided in Supplementary Data S1.

Supplementary Table S1. Exact database strategies

Source and date	Exact strategy / limits
PubMed/MEDLINE; inception to 29 July 2026	("Atrial Fibrillation"[Mesh] OR "atrial fibrillation"[tiab])<br>AND<br>("Heart Failure"[Mesh] OR "heart failure"[tiab] OR HFrEF[tiab] OR HFmrEF[tiab] OR HFpEF[tiab] OR "heart failure with reduced ejection fraction"[tiab] OR "heart failure with mildly reduced ejection fraction"[tiab] OR "heart failure with preserved ejection fraction"[tiab] OR "heart failure with improved ejection fraction"[tiab] OR "arrhythmia-induced cardiomyopathy"[tiab] OR "tachycardia-induced cardiomyopathy"[tiab])<br>AND<br>("Catheter Ablation"[Mesh] OR "Anti-Arrhythmia Agents"[Mesh] OR "Cardioversion, Electric"[Mesh] OR "Anticoagulants"[Mesh] OR "Cardiac Resynchronization Therapy"[Mesh] OR "rhythm control"[tiab] OR "early rhythm control"[tiab] OR "rate control"[tiab] OR antiarrhythmic*[tiab] OR cardioversion[tiab] OR ablation[tiab] OR "pulmonary vein isolation"[tiab] OR cryoballoon[tiab] OR "radiofrequency ablation"[tiab] OR "pulsed field ablation"[tiab] OR "pulsed-field ablation"[tiab] OR "posterior wall isolation"[tiab] OR "atrioventricular node ablation"[tiab] OR "atrioventricular junction ablation"[tiab] OR "cardiac resynchronization therapy"[tiab] OR anticoagula*[tiab])<br>AND<br>(randomized controlled trial[pt] OR controlled clinical trial[pt] OR meta-analysis[pt] OR systematic review[pt] OR practice guideline[pt] OR guideline[pt] OR consensus[tiab])<br>NOT<br>(animals[mh] NOT humans[mh])
Cochrane Library (CDSR and CENTRAL); inception to 29 July 2026	"atrial fibrillation"<br>AND<br>("heart failure" OR HFrEF OR HFmrEF OR HFpEF OR "heart failure with reduced ejection fraction" OR "heart failure with mildly reduced ejection fraction" OR "heart failure with preserved ejection fraction" OR "heart failure with improved ejection fraction" OR "arrhythmia-induced cardiomyopathy" OR "tachycardia-induced cardiomyopathy")<br>AND<br>("rhythm control" OR "early rhythm control" OR "rate control" OR antiarrhythmic* OR cardioversion OR ablation OR "pulmonary vein isolation" OR cryoballoon OR radiofrequency OR "pulsed field ablation" OR "pulsed-field ablation" OR "posterior wall isolation" OR "atrioventricular node ablation" OR "atrioventricular junction ablation" OR "cardiac resynchronization therapy" OR anticoagula*)
OpenAlex; 1 January 1900 to 29 July 2026	title_and_abstract.search:"atrial fibrillation" AND ("heart failure" OR HFrEF OR HFmrEF OR HFpEF OR "heart failure with reduced ejection fraction" OR "heart failure with mildly reduced ejection fraction" OR "heart failure with preserved ejection fraction" OR "heart failure with improved ejection fraction" OR "arrhythmia-induced cardiomyopathy" OR "tachycardia-induced cardiomyopathy") AND ("rhythm control" OR "early rhythm control" OR "rate control" OR antiarrhythmic OR cardioversion OR ablation OR "pulmonary vein isolation" OR cryoballoon OR radiofrequency OR "pulsed field ablation" OR "pulsed-field ablation" OR "posterior wall isolation" OR "atrioventricular node ablation" OR "atrioventricular junction ablation" OR "cardiac resynchronization therapy" OR anticoagulation) AND (randomized OR randomised OR trial OR "systematic review" OR "meta-analysis" OR guideline OR consensus),from_publication_date:1900-01-01,to_publication_date:2026-07-29,language:en,is_retracted:false
Targeted update; 30 July 2026	Separate topic-specific searches combined "atrial fibrillation" and, where relevant, "heart failure" with device, trial, technique, phenotype, monitoring, quality-of-life, patient-perspective, access, risk-factor, and ongoing-study terms. ClinicalTrials.gov was searched by trial name and NCT identifier. These supplementary searches did not constitute a single countable database query.

OpenAlex API reproducibility note

The dated OpenAlex record set in Table S2 used the field-restricted title_and_abstract.search filter shown in Table S1. This syntax remains operational but is now labelled as a legacy filter in the OpenAlex documentation, which recommends the separate search= parameter. The archival syntax is retained so the dated scope is not relabelled retrospectively.

Current prospective rerun parameters (checked 31 July 2026): endpoint=https://api.openalex.org/works; search=[the complete three-block Boolean string in Table S1 and Supplementary Data S1]; filter=from_publication_date:1900-01-01,to_publication_date:2026-07-30,language:en,is_retracted:false; per_page=100; cursor=*; select=id,doi,ids,title,publication_year,publication_date.

A fully URL-encoded executable GET link, the exact archival filter, the current parameter file, dated JSON/CSV exports, and checksums are supplied in Supplementary Data S1. Current search= spans titles, abstracts, and available full text; it is therefore a sensitivity and future-update specification, not a field-identical replacement for the dated title-and-abstract set, and it was not used to recalculate Table S2.

Supplementary Table S2. Dated search yields and deduplication

Source or step	Search date	Records	Handling / interpretation
PubMed/MEDLINE	29 July 2026	819	Result of the exact filtered strategy in Table S1.
OpenAlex	29 July 2026	3,744	Records after within-source deduplication.
OpenAlex records matched to PubMed	29 July 2026	558	Matched by PMID, DOI, or normalized title.
Combined unique PubMed + OpenAlex	29 July 2026	4,005	819 + 3,744 − 558; before relevance prioritization.
Cochrane Database of Systematic Reviews	29 July 2026	5	CDSR review results.
CENTRAL	29 July 2026	1,454	1,423 English-language records. Retained separately because complete record-level export and cross-database deduplication required institutional authentication.

Note: Database indexes are dynamic. Verification reruns on 30 July 2026 returned 821 PubMed records and 3,864 OpenAlex records with the same publication-date cutoff and archival field-restricted OpenAlex syntax. The rerun identified 561 PubMed–OpenAlex matches and 4,124 combined unique records. These machine-readable verification files are retained in Supplementary Data S1, while the 29 July source yields above remain the dated retrieval set described in the manuscript. The broader current OpenAlex search= sensitivity query was not substituted into this count.

Contextual-evidence note. The 2026 atrial-cardiomyopathy review (Pierucci et al.; DOI: 10.23736/S2724-5683.25.06725-0) was added as contextual evidence after the dated database searches. It was not included in the retrieval counts in Supplementary Table S2 and was not treated as intervention-effect evidence.

Supplementary Table S3. Narrative evidence scope and handling

Domain	Approach used for the narrative synthesis
Population	Adults with AF and HFrEF, HFmrEF, HFpEF, heart failure with improved EF, advanced HF, or suspected AF-/arrhythmia-mediated cardiomyopathy; extractable HF subgroups were considered.
Management topics	Rate and rhythm control, antiarrhythmic drugs, cardioversion, catheter ablation, PVI and additional lesion sets, thermal and pulsed-field energy sources, AV-junction ablation with pacing, CRT, anticoagulation, rhythm monitoring, and relevant HF therapy.
Clinical outcomes	Mortality, HF hospitalization, stroke/systemic embolism, AF recurrence or burden, LVEF, exercise capacity, symptoms, quality of life, patient-reported outcomes, treatment burden, and procedural safety.
Evidence prioritization	Direct randomized evidence in the target phenotype was given the greatest interpretive weight. Prespecified randomized subgroups were distinguished from post hoc analyses. Observational, single-arm, and pilot studies were used mainly when direct evidence was unavailable or for safety, feasibility, or hypothesis generation. Guidelines and consensus documents informed practice context; trial registries described study design and status only.

Reports not used as principal evidence	Pediatric and preclinical studies; case reports; small uncontrolled series without adequate comparative information; abstracts without sufficient outcome data; duplicates; and reports without an AF–HF population or extractable HF subgroup.
Narrative synthesis	Evidence was labelled as direct randomized, prespecified randomized subgroup, post hoc subgroup, observational or single-arm, pilot, guideline/consensus, or author-derived interpretation. These labels indicate evidentiary provenance and do not constitute a formal GRADE assessment. Study selection was purposive; duplicate independent screening, protocol registration, PRISMA-style study selection, quantitative pooling, and formal risk-of-bias assessment were not performed.

Abbreviations: AF, atrial fibrillation; AV, atrioventricular; CDSR, Cochrane Database of Systematic Reviews; CENTRAL, Cochrane Central Register of Controlled Trials; CRT, cardiac resynchronization therapy; DOI, digital object identifier; EF, ejection fraction; HF, heart failure; HFmrEF, heart failure with mildly reduced ejection fraction; HFpEF, heart failure with preserved ejection fraction; HFrEF, heart failure with reduced ejection fraction; LVEF, left ventricular ejection fraction; PMID, PubMed identifier; PVI, pulmonary vein isolation.
